# Update on musculoskeletal applications of magnetic resonance-guided focused ultrasound

**DOI:** 10.1007/s00256-024-04620-8

**Published:** 2024-02-16

**Authors:** Kevin C. McGill, Joe D. Baal, Matthew D. Bucknor

**Affiliations:** Department of Radiology and Biomedical Imaging, University of California, San Francisco, 505 Parnassus Ave, Suite M391, San Francisco, CA 94143 USA

**Keywords:** Osteoid osteoma, Desmoid tumor, Facet arthropathy, Bone metastases, Magnetic resonance-guided focused ultrasound, High-intensity focused ultrasound, Thermal ablation

## Abstract

Magnetic resonance-guided focused ultrasound (MRgFUS) is a noninvasive, incisionless, radiation-free technology used to ablate tissue deep within the body. This technique has gained increased popularity following FDA approval for treatment of pain related to bone metastases and limited approval for treatment of osteoid osteoma. MRgFUS delivers superior visualization of soft tissue targets in unlimited imaging planes and precision in targeting and delivery of thermal dose which is all provided during real-time monitoring using MR thermometry. This paper provides an overview of the common musculoskeletal applications of MRgFUS along with updates on clinical outcomes and discussion of future applications.

## Introduction

High-intensity focused ultrasound technology works by focusing ultrasound waves to converge on a specific point creating a rise in temperature at the target. When used with magnetic resonance guidance, visualization of the target, often soft tissue, can be better than traditional computed tomography or fluoroscopy-guided interventions. Magnetic resonance-guided focused ultrasound (MRgFUS) facilitates the creation of 3D models which can be useful for pretreatment planning in ablation of soft tissue and osseous lesions [1]. The efficacy and safety of the treatment are also bolstered by real-time temperature monitoring of the target and surrounding tissue through magnetic resonance thermometry, which enables real-time ablation energy adjustments to maximize the desired treatment effect [2]. This innovative treatment has led to a paradigm shift in the approach to a variety of diseases across many fields of medicine.

Radiofrequency ablation and cryoablation have long been a standard treatment in the field of minimally invasive thermal ablation [2, 3]. These techniques, however, require a tiny probe to enter the body which can potentially damage adjacent tissue, even when successful [3, 4]. The ability to ablate small areas without damaging the adjacent tissue made MRgFUS ideal in treatment of soft tissue masses for which radiation therapy and/or surgical intervention could pose a significant risk of morbidity.

The first musculoskeletal application of MRgFUS to gain popularity was palliation of persistently painful bone metastases following radiotherapy, an indication which has shown favorable outcomes [5, 6]. Now other indications are being investigated with promising results such as treatment of osteoid osteoma, facet arthropathy, and desmoid tumors. The purpose of this paper is to describe the musculoskeletal uses for MRgFUS and provide an update on the recent advancements in the field along with clinical outcomes and a brief discussion of future musculoskeletal and non-musculoskeletal applications.

## MRgFUS techniques

### Basic principles

MRgFUS systems use high-energy ultrasound transducers in phased arrays to convert electric signals into sound waves with a typical frequency range of 200 kHz to 4 MHz and acoustic intensity between 100 and 10,000 W/cm2. These devices typically can raise local tissue temperature to 65 to 85 °C in approximately 20 s [7].

### Pre-procedure considerations

Pre-procedure coordination with the anesthesia team is required to ensure the patient’s plan for anesthesia and pain control after the procedure is fully optimized. Since many anesthesiologists have not participated in an MRgFUS case before, early communication is essential to help make sure they are familiar with challenges unique to MRgFUS cases such as patient positioning and expectations regarding pain management. It is difficult to generalize regarding pain management requirements as these are highly dependent on the structure being targeted, the size of the target lesion, and the proximity to nerves. Combinations of monitored care with sedation, regional anesthesia, and general anesthesia can all be used with success. At our institution, we perform all treatments under general anesthesia, occasionally supplementing with regional anesthesia if significant post-operative pain is anticipated [8].

### Treatment devices

The only MRgFUS devices which have received FDA premarket approval (PMA) are the ExAblate devices manufactured by Insightec (Insightec Ltd; Haifa, Israel) which currently works on GE scanners (GE Healthcare; Waukesha, WI). The Insightec in-table transducer system was approved for uterine fibroids in 2004 and for bone metastases in 2012. The Sonalleve device manufactured by Profound Medical (Mississauga, ON, Canada) works with Philips MRI scanners (Amsterdam, Netherlands) and received a Humanitarian Device Exemption approval for treatment of osteoid osteomas in 2020. Both devices offer similar functionality and no further distinction between them will be noted for this review.

### Positioning and safety

After patient preparation, the patient must be transferred to the treatment table with the in-table transducer located underneath the area of the body that is being treated and carefully secured into place with Velcro straps. Additional padding is essential to prevent pressure injuries and possible neuropraxias. Careful coordination with available patient positioning devices from the medical center’s operating room is helpful to optimize treatment safety as standard MRI patient tables generally have inadequate padding for the length of time the patient will be positioned on the table under anesthesia.

The risk of skin injury can be significant depending on the proximity of the lesion to the skin and the amount of energy being used, which is generally higher for soft tissue tumor treatments compared to bone tumor treatments. The near-field skin, where sound enters the patient, can be protected with cold-degassed water poured into a shallow depression at the interface between the patient and the gel pad. Planning MR images should be carefully examined for the presence of air bubbles along the skin interface because sound energy can focally absorb at these interfaces. More degassed water can be poured into the near field in order to displace these air bubbles [9].

There is also the possibility for sound energy to propagate to the far-field skin, even if there is significant absorption at the target. Sound energy reaching this location will reflect at the air-skin interface and can lead to significant thermal injury. A coupling device in this location such as a cold-water bag or gel pad can help to dispel sound energy and directly cool the skin.

### Treatment planning and monitoring

**Fig. 1:**
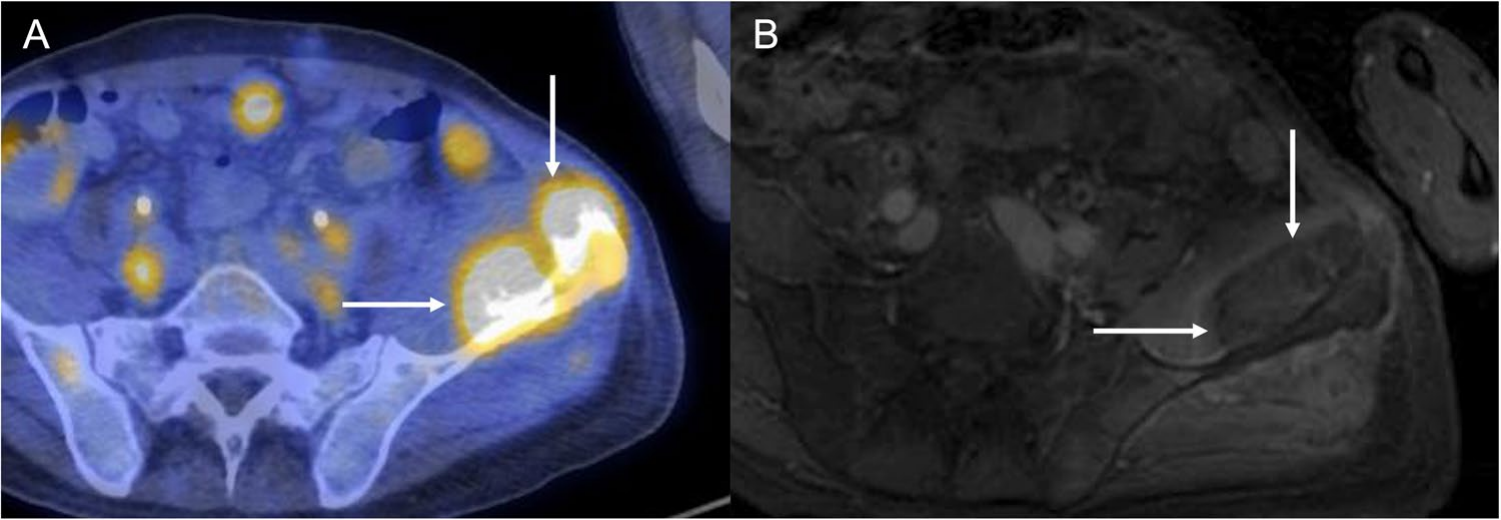
Osseous metastasis. **A** Axial fused pre-treatment FDG PET/CT of the left iliac bone in a patient with metastatic clear cell sarcoma demonstrating hypermetabolism (white arrows) within the bone and adjacent soft tissue. **B** Post-treatment axial T1 post-contrast images demonstrate relative hypoenhancement within the ablation area with minimal rim enhancement

Planning sequences are obtained in the axial, coronal, and sagittal orientations. T1-weighted or T2-weighted fat-saturated imaging may be preferred depending on how well the target lesion is seen on a given sequence. These sequences are then transferred to a dedicated workstation with software tools that allow the delineation of the skin, critical structures, bone, and the target lesion/region of treatment (Fig. 1).

Next, test sonications are performed that allow the system to calibrate geometry and thermal dose. After this step, the software proposes a set of sonications to thermally ablate the target and multiple parameters can be adjusted and optimized including energy level, beam angle, transducer frequency, and sonication density, along with the number of sonications. Treatment monitoring is performed using MR thermometry sequences which demonstrate the change in temperature from the beginning to the end of the sonication. The total time for the cycle through each individual sonication lasts approximately 60 to 90 s. The size of the ablation per sonication can typically be as small as a sphere approximately 0.5 cm in diameter or as large as a cylinder 4–5 cm in length and 1.5 cm in diameter. Calculating ideal sonication morphometry is an ongoing area of active research.

After all planned sonications are completed, a T2-weighted fat-saturated sequence can demonstrate post-procedure edema which can be helpful in anticipating the patient’s pain management needs. Pre- and post-contrast T1-weighted fat-saturated imaging is also typically obtained to evaluate nonperfused volume which approximates the percentage of tumor ablated.

## Painful bone metastases

### Overview

Because of decreasing cancer mortality rates due to improved cancer diagnosis, treatment, and management, there is an increasing population of patients who live with cancer comorbidities [10, 11]. For many types of malignancy, especially breast and prostate cancer, the bone is the most frequent site of metastasis. Symptomatic bone metastases are a substantial contributor to cancer-related pain with up to 67% of affected patients experiencing moderate to severe pain [12, 13]. Bone metastases typically indicate disseminated disease and short-term prognosis with a median survival of up to 48 months [14]. As such, patients with painful bone metastases are often treated with palliative intent with a focus on improving quality of life, including management of cancer-related pain. Currently, the standard palliative treatment for painful bone metastases includes analgesics, including opioid medications, and noninvasive procedures like external beam radiation therapy (EBRT) [15]. However, opioid analgesics are limited by medication side effects and EBRT only achieves approximately 60–70% treatment response rate [16]. EBRT is also limited by radiation dose limits and various adverse effects such as insufficiency fractures. As a result, patients with painful bone metastases may be globally undertreated for their pain [17]. To help bridge this gap, MRgFUS has gained recognition in recent years as a safe and effective noninvasive treatment method for symptomatic bone metastases [18–20].

### Safety and efficacy

In the last two decades, numerous clinical studies have showcased substantial efficacy of MRgFUS in treating painful bone metastases. A recent meta-analysis of over 30 studies with over 1000 patients with painful bone metastases treated with MRgFUS demonstrated complete or partial pain relief in approximately 79% of patients while achieving rates of low-grade and high-grade treatment-related adverse events at less than 6% and 1%, respectively [18]. Moreover, pain scores progressively decreased up to several months following treatment, suggestive of favorable long-term pain palliation. Another meta-analysis demonstrated decreased usage of pain medication from baseline and follow-up following MRgFUS treatment, another important index of pain relief [19].

MRgFUS treatment response rates are similar to those seen with EBRT (ranging 60–80%). In a matched-pair study comparing MRgFUS and radiation therapy, MRgFUS was found to provide pain palliation within 1 week, and no significant difference in overall pain scores was observed after 1–2 months [21]. However, unlike radiation therapy, radiation dose limits do not impose restrictions on the number of MRgFUS treatment sessions a patient can undergo. Therefore, MRgFUS serves as a viable treatment alternative to provide pain relief in patients who are refractory to prior radiation therapy and to overcome challenges with cancers exhibiting radioresistance [5, 22, 23]. Currently, the synergistic effect of combined radiation and high-intensity focused ultrasound therapy on painful bone metastasis is not well understood and remains an area of ongoing research [24].

### Technical considerations

The hypothesized mechanism of pain palliation from MRgFUS is from thermal denervation of periosteum, which was previously demonstrated in preclinical histologic analyses [25]. Since bone has the propensity to absorb nearly 50 times more acoustic energy than soft tissue, lower energy levels may suffice to achieve efficient thermal ablation for bone lesions [26]. A recent study found that higher energy density applied to the bone surface is predictive of pain relief following MRgFUS treatment [27]. Intra-operative monitoring of the amount of applied energy density may facilitate optimal treatment efficacy. Current treatment protocols can also employ MR-based proton resonance frequency (PRF) thermometry of surrounding soft tissues to provide near real-time temperature information which can be used to predict treatment efficacy [28].

Typically, MRgFUS is most suited for localized bone metastasis in the non-articular appendicular skeleton and the posterior aspects of the sacral, lumbar, and thoracic spine. For optimal safety, targeted lesions should be at least 1 cm from nerve bundles, joint spaces, vasculature, and the skin surface [29]. Fracture risk should be assessed (e.g., Mirels’ Classification) during the patient selection process [30]. In terms of treatment planning, a “direct” approach places the center of sonication at the bone-soft tissue interface, which allows for maximal energy deposition and a more focused area of ablation [31, 32]. Alternatively, positioning the center of the sonication deep to the bone surface allows for a larger ablation area but with decreased energy density [33].

## Osteoid osteomas

### Overview

**Fig. 2:**
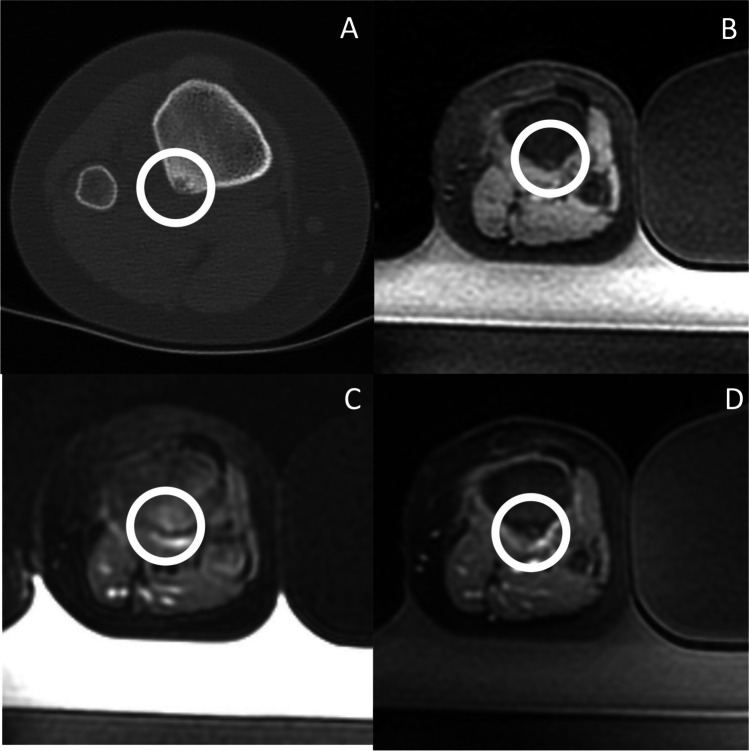
Osteoid osteoma. **A** Axial CT image in a patient who developed recurrent night pain relieved with NSAIDS approximately 10 months after initially successful CTgRFA for osteoid osteoma. The circle demonstrates an area of persistent subcortical lucency consistent with recurrent or residual nidus. **B** Pre-treatment axial T1-weighted FSPGR image demonstrates slight hyperintensity at the location of the nidus, highlighted by the circle. **C** Pre-treatment axial T2 FS image demonstrates subtle bone marrow edema pattern and overlying soft tissue edema at the site of the nidus, highlighted by the circle. **D** Post-treatment axial T1-weighted FSPGR post-contrast image demonstrates mild hypoenhancement at the treated nidus and mild hyperenhancement of the overlying soft tissues, highlighted by the circle. The patient’s pain completely resolved the day following the treatment

Osteoid osteomas are painful benign bone tumors that typically occur in the cortices of the long bones of children and adolescents, most frequently involving the femur and the tibia, accounting for 10% of all benign bone tumors [34]. Percutaneous computed tomography-guided radiofrequency ablation (CTgRFA) has replaced surgery as the standard of care because of a > 90% efficacy and favorable side effect profile. In recent years, MRgFUS has emerged as a safe and effective approach for ablation of osteoid osteomas (Fig. 2).

### Efficacy and technique

Several clinical trials have demonstrated similar efficacy of MRgFUS when compared to CTgRFA for osteoid osteoma treatment. A prospective multi-institutional trial of MRgFUS treatment of 29 patients with osteoid osteoma found the treatment to have a 90% response rate [35]. More recently, a small non-randomized retrospective study involving 30 patients also found a similar response rate compared to CTgRFA [4]. Additionally, a recent meta-analysis of 113 studies published between 2012 and 2022 with a total sample size of 353 patients found a success rate of 92.8% (95% CI, 89.8–95.7%) and the incidence of minor complications (thermal injury at the ablation site) to be 0.85% [36].

### Pearls and pitfalls

Patient selection is critical as MRgFUS, unlike CTgRFA, does not offer the opportunity to obtain a biopsy sample to confirm the diagnosis. Common mimics of osteoid osteomas include subacute osteomyelitis (Brodie’s abscess) and musculoskeletal injuries where tendons or muscles are connecting directly to the bone, for example, adductor insertion avulsion syndrome.

It can be difficult to visualize the nidus during treatment secondary to its small size, particularly depending on slice thickness, slice spacing, and volume averaging. 3D T1-weighted fast spoiled gradient echo sequences with fat saturation are commonly used at our institution to improve visualization of the nidus (which is typically hyperintense on this sequence) with thin slices and relatively shorter acquisition times.

At our institution, we most commonly use approximately six sonications for ablation of the osteoid osteoma nidus. There are techniques that can be used to improve penetration of sound energy beyond the cortical surface, including repetition of a sonication or lengthening of the individual sonication duration. However, less commonly some osteoid osteomas with markedly thick periosteal reaction or an intramedullary location may prevent sound energy from reaching the nidus. These patients may be less amenable to treatment with MRgFUS.

## Facet arthropathy

### Overview

Chronic low back pain (LBP) is a common cause of disability which can have a lifetime prevalence as high as 84% [37]. Facet (zygapophyseal) osteoarthritis represents a sizable segment of the population suffering with LBP with 15–45% of LBP patients attributing the pain to the facets [38–40]. If conservative treatments, including activity modification, oral analgesics, and physical therapy, fail, the next step may be intra-articular injection of local anesthetics and/or corticosteroids. In addition to providing pain relief, injections can be diagnostic, confirming the anatomic location within the spine and the vertebral level. While studies have proven injections to be effective, like many other joint or nerve injections, they often only provide temporary relief and may need to be repeated periodically in 3- to 4-month intervals [41, 42].

Facetogenic pain is thought to be mediated by the pain in the joint capsule, supplied by the medial branch nerve arising from the dorsal ramus. Some interventions are therefore designed to disrupt the nerve supply by either directly targeting the median branch of the dorsal ramus or the nerve endings of the posterior facet capsule. Traditionally, this has been done by focusing thermal ablation on the medial branch nerve minimally invasively with CTgRFA. The technique for CTgRFA, in which the probe is directed to the junction of the transverse process and the superior articular facet, has been proven moderately effective for long- and short-term pain relief [41, 43]. The noninvasive and radiation-free MRgFUS ablation of the facets has also shown promising results [44–46].

### Efficacy and technique

Due to the slightly different targets, the use of CTgRFA and MRgFUS for facetogenic pain has the potential to be complementary. Phantom studies have shown that the area can be appropriately targeted with FUS [47]. Animal studies have also proven safety and the ability to achieve thermal necrosis [48]. In 2022, Perez et al. published a small pilot study of noninvasive fluoroscopy-guided FUS with 10 participants using the same landmarks as CT, reporting treatment success of 90%, 50%, 60%, and 40% at 1, 3, 6, and 12 months, respectively, which demonstrated overall results resembling those of CTgRFA [49]. A recent retrospective study by Tiegs-Heiden et al. on safety and tolerability of MR-guided FUS in 20 patients (26 treatments) who failed CTgRFA showed that 57.1% reported pain relief for more than 3 months and 80% of patients with previous improvement with CT reported improvement with MR [45].

### Pearls and pitfalls

The main drawbacks of MRgFUS are primarily related to the MR component of the procedure. There are limitations in patient size due to MR bore restrictions and treatment table weight limits as low as 250 lbs [45]. The high cost, limited insurance coverage, and the onerous safety checks surrounding MR-guided procedures are also barriers. Furthermore, patients who have non-conditional implanted devices may not be candidates for the procedure. While an MRgFUS has been successfully used to treat facetogenic LBP in at least one patient with a non-conditional cardiac device, this requires additional personnel including cardiac nurse and an MR physicist to be present during the procedures [50].

MR is better than CT at depicting spinal anatomy and allows a greater number of imaging planes [51]. While the treatment planning is more complex in MRgFUS and more time-consuming compared to CTgRFA, this planning is crucial to maintaining the safety of the procedure.

## Desmoid tumors

### Overview

**Fig. 3:**
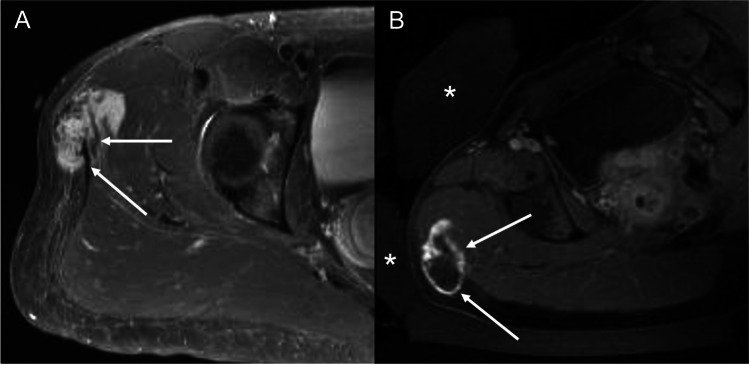
Desmoid tumor. **A** Pre-treatment axial T1-weighted fat-saturated post-contrast sequence obtained prior to MRgFUS demonstrates a homogeneously enhancing mass in the lateral hip superior to the greater trochanter, compatible with a biopsy-proven desmoid tumor (white arrow). **B** Post-treatment axial T1-weighted fat-saturated post-contrast sequence performed immediately following MRgFUS exhibits 85% ablation area with minimal residual rim enhancement (white arrows). Water bags (asterisks) are positioned anterior and lateral to the right hip to minimize the risk of skin injury

Desmoid tumors are rare soft tissue tumors resulting from myofibroblastic tissue proliferation with an annual incidence of approximately 2–4 per one million people [52]. While histologically benign, desmoid tumors tend to be locally aggressive with an infiltrative growth pattern and propensity for recurrence after treatment [53]. Associated symptoms may be debilitating and can vary based on the affected organs and adjacent structures. The first-line treatment for desmoid tumors is surgical resection, which has recurrence rates ranging up to 50% even when negative margins are achieved [54]. Adjuvant radiation therapy can achieve more favorable local tumor control ranging between 70 and 80% but is limited by radiation-induced morbidity observed in up to 30% of patients, which includes pathologic fractures and development of secondary malignancies, particularly among younger patient populations [55]. Adjuvant systemic chemotherapy, namely tyrosine kinase inhibitors such as sorafenib, has an overall treatment response rate of 33% but is associated with moderate to severe complications in up to 47%, which are predominantly skin disorders [56]. Given the less than favorable safety profile of surgery and adjuvant chemoradiotherapy, as well as the potential need for repeated treatments, the approach to managing symptomatic desmoid tumors has expanded in recent years to include noninvasive treatments like MRgFUS as a primary, adjuvant, or salvage treatment of desmoid tumors [57] (Fig. 3).

### Efficacy and safety

In a recent multi-center study involving 105 patients, subtotal treatment of extra-abdominal desmoid tumors resulted in approximately 34% decrease in total tumor volume and 64% decrease in viable tumor volume following MRgFUS treatment. About 86% of treated patients were found to have either stable or decreased disease burden at follow-up with a median progression-free survival of 17 months [3]. The overall side effect rate was 36% and was predominantly mild skin burns. The relatively favorable safety profile of MRgFUS allows for repeated treatments without the added concern of radiation dose limits or risk of secondary malignancy [58]. To minimize the risk of skin burns, a shallow cool water bath can be applied to the near-field skin and cool water bags and gel pads can be placed along the far-field skin [9, 59]. A fiberoptic temperature probe may also be used for real-time temperature monitoring and feedback during treatments [58]. Periodic skin inspection and intermittent ice cooling strategy throughout the treatment period should be considered especially along the far-field skin where there is limited evaluation for heating with MR thermometry in certain extra-abdominal regions [9].

As with other conditions treated with MRgFUS, the potential for thermal damage to neighboring vital soft tissue structures, such as blood vessels and nerves, plays a significant role in determining treatment intent and feasibility. If a desmoid tumor is sufficiently distant from vital soft tissues, MRgFUS may be utilized with curative intent to completely ablate tumor volume. When the tumor closely approximates vital structures, MRgFUS can be used for subtotal treatment with the intent of controlling tumor growth. However, in a paper on early clinical experiences with desmoid tumor, investigators demonstrated that a successful ablation can be achieved even when a vital structure such as the sciatic nerve courses through the lesion, which would be difficult to accomplish with other ablation techniques [58].

Overall, MRgFUS seems to be a moderately effective therapy for symptomatic desmoid tumors that can achieve considerable local tumor control even with subtotal treatments and is a viable treatment option to supplement chemoradiation [3, 58, 60].

## Other indications/future uses

MRgFUS is increasing in use in the musculoskeletal field and has been investigated for treating pain related to knee osteoarthritis and vascular malformations [59, 61]. An international multicenter randomized controlled trial known as the Focused Ultrasound and Radiotherapy for noninvasive palliative treatment of bone metastasis (FURTHER) study is an ongoing project which aims to evaluate the effectiveness and cost-effectiveness for MRgFUS with and without external beam radiotherapy for the treatment of cancer-related bone pain [24]. MRgFUS has been investigated as a treatment for soft tissue sarcomas [62] and may have an effect on primary bone malignancy [63] in addition to the palliative effect. Promising growth has been demonstrated in treatment of a variety of neurologic conditions [64, 65], especially for essential tremor [66, 67]. Some additional non-musculoskeletal lesions MRgFUS may be useful for include pancreatic cancer [68], prostate cancer [69], and thyroid nodules [70].

## Conclusions

MRgFUS is a useful tool for treating a variety of musculoskeletal ailments. By focusing ultrasound waves, this technology can be used noninvasively to ablate osseous and soft tissue structures to treat both benign and malignant conditions. The most common musculoskeletal indications for MRgFUS are painful bone metastases, osteoid osteoma, facet arthropathy, and desmoid tumor. Non-musculoskeletal uses are also advancing primarily for treatment of neurologic conditions with the most promising clinical results in the management of essential tremor and Parkinson’s disease. The ability of MRgFUS to alter permeability of the BBB generates more potential for development of both adjuvant and novel treatments for intracranial pathology. MRgFUS is a promising emerging technology which continues to expand its utility throughout the field of medicine.
